# Enhanced expression of *recX* in *Mycobacterium tuberculosis* owing to a promoter internal to *recA*

**DOI:** 10.1016/j.tube.2010.11.002

**Published:** 2011-03

**Authors:** Lorna N. Forse, Joanna Houghton, Elaine O. Davis

**Affiliations:** Division of Mycobacterial Research, MRC National Institute for Medical Research, The Ridgeway, Mill Hill, London NW7 1AA, UK

**Keywords:** *Mycobacterium tuberculosis*, RecX, Intragenic promoter

## Abstract

RecX is a small protein that interacts with, and modulates the activity of, RecA protein. In mycobacteria the *recX* gene is located immediately downstream of the *recA* gene, and the coding regions overlap. It has previously been shown that these two genes are co-transcribed in *Mycobacterium smegmatis*. In this study we examine the expression of *recX* in *Mycobacterium tuberculosis*. In addition to being co-transcribed with *recA* from the DNA-damage inducible *recA* promoters, we identify a constitutive *recX* promoter located within the *recA* coding sequence that is strong enough to make a significant contribution to the expression level of *recX* in the absence of DNA damage. Intriguingly, this promoter is inactivated in *M. smegmatis* by a critical base change in the −10 promoter motif, which probably accounts for the lower level of expression of *recX* relative to *recA* that we observed in that species. It is possible that this difference in relative expression influences RecA functions including the response to DNA damage of LexA-regulated genes.

## 1 Introduction

The RecX protein was first identified as being encoded by a small open reading frame downstream of the *recA* gene in *Pseudomonas aeruginosa*^1^ and has since been found to be conserved in many bacterial species.^2,3^ The RecA protein is the key protein involved in homologous genetic recombination and recombinational DNA repair.^4^ RecA also plays a central role in regulation of gene expression in response to DNA damage by the repressor LexA via its stimulation of LexA cleavage on binding to regions of single-stranded DNA.^5^

A role for RecX as a negative regulator of RecA was initially indicated by data demonstrating that the RecX protein was necessary to overcome the harmful effects of RecA over-expression in bacterial species as diverse as *P. aeruginosa*,^1^ *Mycobacterium smegmatis*,^6^ *Streptomyces lividans*^7^ and *Xanthomonas oryzae* pv *oryzae*.^8^ Subsequently, the RecX protein was shown to inhibit the ATPase, recombinase and co-protease activities of RecA.^9,10^ Detailed studies in *Escherichia coli* revealed that RecX acts by binding to the end of the growing RecA filament, thus limiting filament growth and leading to net disassembly.^11^ RecX is also able to bind within the major helical groove of the RecA filament at high relative concentrations which would block strand-exchange.^12^ Interestingly, in *Deinococcus radiodurans*, RecX also acts as a negative regulator of *recA* expression, with deletion of the *recX* gene resulting in constitutive activation of the *recA* promoter and over-expression of RecX suppressing *recA* promoter activity.^13^

In most species studied to date *recX* is found downstream of the *recA* gene, and in a number of cases the two genes are co-transcribed, e.g. in *S. lividans*,^7^ *M. smegmatis*^14^ and *E. coli*.^15^ However, in *Xanthomonas* pathovars, the genes in the conserved *lexA-recA-recX* loci are each expressed from their own promoter.^16^ Exceptions to the typical *recA-recX* genomic organisation include bacterial species such as *Bacillus subtilis* and *Neisseria gonorrhoeae* in which the *recA* and *recX* genes are separated on the chromosome by distances as great as 838 kb and 275 kb respectively.^9,17^

In a number of mycobacterial species, as well as a few other bacteria,^2^ the 5′ part of the *recX* coding sequence overlaps the 3′-region of the *recA* gene. In *M. smegmatis*^14^ the overlap is 32 bp long, while in *Mycobacterium tuberculosis*,^18^ *Mycobacterium leprae*^19^ *Mycobacterium avium* and *Mycobacterium ulcerans* a 35 bp overlap is present. The difference between the *M. smegmatis* sequence and those of the others is equivalent to the loss of a single codon rather than three separate base deletions. Among mycobacteria *M. smegmatis* has the simplest *recA-recX* locus arrangement, with the two genes flanked by genes expressed from the complementary DNA strand. In *M. tuberculosis*, *recA-recX* is followed by a single gene in the same orientation, termed *Rv2735c*, classified as encoding a conserved hypothetical protein with no known function. A further difference between *M. tuberculosis* and *M. smegmatis* in this region is the presence of an intein in *M. tuberculosis* but not *M. smegmatis recA*^20^; an intein is an intervening sequence that is removed post-translationally in a process termed protein splicing.^21^

The co-transcription of *recA* and *recX* has already been described in *M. smegmatis*.^14^ In this study we show that the transcriptional regulation of *recX* in *M. tuberculosis* differs from *M. smegmatis*, with *recX* being co-transcribed with *recA* but at the same time possessing its own promoter, the entirety of which is present within the coding region of the preceding *recA* gene.

## 2 Methods

### 2.1 Bacterial strains, plasmids and growth conditions

*E. coli* strains Alpha-select Silver efficiency (Bioline, London, UK) and DH5α (Invitrogen) were used as host strains for plasmid manipulations. *E. coli* cells were grown at 37 °C on LB agar plates or in LB medium with shaking at 225 rpm; where appropriate antibiotics were added at the following concentrations: ampicillin 100 μg/ml, kanamycin 50 μg/ml. *M. tuberculosis* strains H37Rv, 1424 (a streptomycin resistant derivative of H37Rv^22^ that is the parental strain for the *recA* mutant) and Δ*recA*^22^ were grown at 37 °C on Difco Middlebrook 7H11 agar (Beckton Dickinson) plates supplemented with 4% albumin and 0.5% (w/v) glycerol or in modified Dubos medium (Difco) supplemented with 4% albumin and 0.2% (w/v) glycerol rolling at 2 rpm. Where appropriate, antibiotics were added to *M. tuberculosis* cultures at the following concentrations: kanamycin 25 μg/ml, streptomycin 100 μg/ml, hygromycin 50 μg/ml. All procedures with live *M. tuberculosis* were carried out under ACDP containment level 3 conditions.

### 2.2 Recombinant DNA techniques

Plasmid DNA was prepared using miniprep kits (QIAGEN) as described by the manufacturer. The plasmids used and their construction are described in Table 1, and the primers used in this study are listed in Table 2. The locations of key primers and other features in the *recA*-*recX* sequence are shown in Supplementary figure 1. Reporter constructs were created by cloning PCR fragments generated using the primer pairs indicated in Table 1 into the integrating *lacZ* transcriptional vector pEJ414.^23^ PCR reactions for cloning utilised PfuUltra^®^ Hotstart DNA Polymerase (Stratagene) and buffer; all reactions contained 5% DMSO (Sigma–Aldrich). Site-directed mutagenesis was performed as described in the QuikChange^®^ Site-Directed Mutagenesis Kit (Stratagene) but with two 15-cycle PCR reactions; an extra 1 μl of DNA polymerase was added between reactions. For other DNA manipulations, standard DNA protocols were followed.^24^ For each clone or mutant made, the sequences of the promoter region and the junctions to the vector were determined by commercial sequencing using the Illumina Genome Analysis system (Geneservice). Clones were introduced into *M. tuberculosis* via electroporation as described previously.^25^

### 2.3 RNA preparation and reverse transcription

The FastRNA^®^ Pro Blue Kit (Qbiogene) was used for the isolation of total RNA from *M. tuberculosis* (50–100 ml) grown to OD_600_ of ∼0.6. Contaminating DNA in the RNA preparations was digested using TURBO RNase-free DNase (Ambion), and the RNA was subsequently cleaned up using an RNeasy MiniKit (QIAGEN). RNA concentrations were determined spectrophotometrically at 260 nm. Removal of DNA was confirmed by performing PCR using an aliquot of the DNase-treated RNA as a template. Reverse transcription for qRT-PCR and shorter transcripts used Superscript II (Invitrogen) according to the product information. Reverse transcription for long transcripts used QuantiTect Reverse Transcriptase (QIAGEN) according to the product information but with an extended (2 h) 42 °C incubation time. PCR reactions following reverse transcription (RT-PCR) were performed using REDTaq ReadyMix PCR Reaction Mix incorporating REDTaq DNA polymerase (Sigma–Aldrich) with an initial denaturation step of 95 ^°^C for 5 min, followed by 10 cycles of 94 ^°^C for 1 min, 68 ^°^C for 1 min, 72 ^°^C for 8 min, then 35 cycles of 94 ^°^C for 1 min, 58 ^°^C for 1 min, 72 ^°^C for 8 min, and a final extension step of 72 ^°^C for 20 min.

### 2.4 Real-time PCR

Real-time PCR analysis was carried out on the 7500 Fast Real-Time PCR System (Applied Biosystems) using the PCR master mix containing SYBR green dye (Applied Biosystems). The 20 μl PCRs consisted of PCR master mix, 900 nM concentrations of each primer, and 5 μl of cDNA template. The sequences of the primers used in the real-time PCR are given in Table 2. In each case, the test gene and the normalising gene (sigA) were assayed along with a set of standard samples (genomic DNA), and the amounts of gene-specific mRNA were normalised to the amount of sigA mRNA.

### 2.5 5′ RACE transcriptional start site mapping

RNA ligase-mediated 5′ RACE using the Generacer kit (Version 2.0; Invitrogen) was used to map the transcriptional start site of *recX* according to the manufacturer’s guidelines. To facilitate the identification of a transcript start site, which in bacteria carry a 5′-triphosphate,^26,27^ RNA was first treated with tobacco acid pyrophosphatase (TAP), which hydrolyses this group to a 5′-monophosphate to which an RNA oligonulceotide can then be ligated. Following this ligation step and reverse transcription, the resulting cDNA was amplified using the Generacer 5′ forward primer and the appropriate gene-specific primers (Table 2). The products obtained were cloned into pCR4-Blunt-TOPO (Invitrogen) and between five and eight clones were sequenced.

### 2.6 Preparation of cell-free extracts and β-galactosidase assays

Mycobacterial cultures were grown to mid-exponential phase (OD_600_ 0.6) unless otherwise stated; the bacteria were harvested, washed three times in PBS and cell-free extract prepared using a Ribolyser (Hybaid) and glass beads (150-212 microns, Sigma) as described previously.^28^ The supernatant was filtered through a low-binding Durapore 0.22 μm membrane filter (Ultrafree-MC; Millipore) to ensure complete removal of bacteria before removal from CL3 facilities. Where mitomycin C induction was required, cultures were grown to early exponential phase (OD_600_ 0.3) and were then split into two; one culture was induced with 0.02 μg/ml mitomycin C and the other was an uninduced control. Both were then incubated at 37 °C for 24 h before harvesting and preparing cell-free extract as above. Total protein was assayed using a BCA kit (Pierce); β-galactosidase activity was determined using ortho-nitrophenyl-β-galactoside (ONPG) as substrate in 500 μl reactions as described^28^ and expressed in Miller units per milligram of protein.^29^

## 3 Results

### 3.1 Definition of the *recA* operon

**Figure 1:**
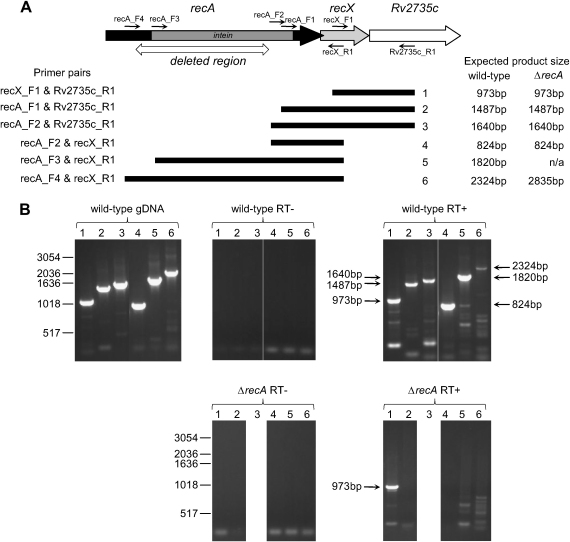
*recX* and Rv2735c are co-transcribed with *recA* in wild-type *M. tuberculosis* but read-through into *recX* is not apparent in the *recA* mutant strain. (A) Schematic showing the relative locations of the three genes *recA*, *recX* and Rv2735c, each depicted by a large arrow. The position of the intein in *recA* is shown by the grey box within the arrow representing *recA*, and the region deleted in *recA* is indicated by the double-headed arrow. The locations of the primers used for RT-PCR analysis are indicated by the small arrows and labeled. The expected PCR products, labeled 1 to 6, formed by the various primer combinations listed on the left are shown by the black bars, with the sizes of these products indicated at the right. (B) The RT-PCR products obtained using wild-type genomic DNA (upper left panel) or RNA isolated from wild-type parental 1424 *M. tuberculosis* (upper middle and right panels) or from the Δ*recA* strain (lower panels). The RT− panels in the centre show the results when reverse transcription was omitted, and the RT+ panels on the right show the products obtained from RNA following a reverse transcription step. The lane numbers correspond to the numbering of the PCR products in (A); PCR 3 was not tested for the Δ*recA* strain. The positions and sizes of the expected PCR products are indicated by the arrows on the right panels, and the positions of DNA molecular mass markers are indicated by the short lines at the left of the gel images.

To define the operon structure of *recA* in *M. tuberculosis*, we assessed co-transcription with the two downstream genes *recX* and Rv2735c by RT-PCR. Initially the presence of RNA spanning each of the pairs of genes *recA*-*recX* and *recX*-Rv2735c was demonstrated by the formation of a product following reverse transcription of RNA isolated from the parental wild-type *M. tuberculosis* strain 1424, but not if the RT step was omitted (Figure 1). To investigate if a single transcript including all three genes was formed, two separate primers within *recA* were used in conjunction with one in Rv2735c, and in each case a product was formed by RT-PCR that was dependent on the reverse transcription step (Figure 1). Thus, *recX* and Rv2735c are at least partially co-transcribed with *recA*.

### 3.2 Expression in the Δ*recA* strain

The mutation in the Δ*recA* strain consists of deletion of 1259 bp between the two *Pst*I sites within the coding sequence for RecA and insertion of a 1770 bp DNA fragment encoding hygromycin resistance.^22^ This mutation would be expected to have a polar effect on the expression of downstream genes that are part of an operon with *recA* owing to the presence of a transcriptional terminator within the fragment inserted.^30,31^ Therefore, we evaluated the expression of *recA*, *recX* and Rv2735c in the parental wild-type strain 1424 and the *recA* mutant by quantitative RT-PCR with normalisation to the housekeeping gene *sigA*.

**Figure 2:**
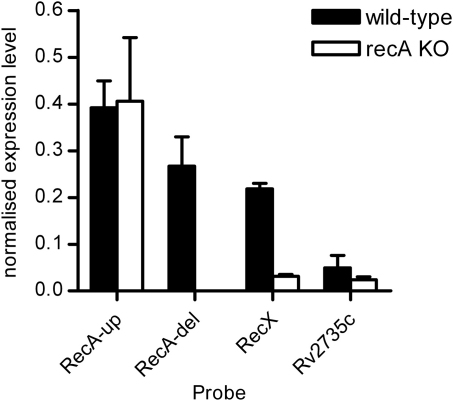
*recX* and Rv2735c are still expressed in the Δ*recA* strain, although at a lower level than in the wild-type. qRT-PCR was performed as described in the Methods using RNA isolated from wild-type *M. tuberculosis* (black bars) or the *recA* mutant (white bars). Expression levels were normalised to that of the housekeeping gene *sigA*. Primer set RecA-up was located in *recA* upstream of the deleted region, while primer set RecA-del was located within the deleted region. Primer sets RecX and Rv2735c were within the coding sequences of the respective genes. Values are the means from three independent cultures, each of which was assayed in triplicate; error bars show standard deviations.

When a primer set located in the part of the *recA* coding sequence remaining upstream of the deletion was used, there was no significant difference between the two strains in the expression level observed, while, as expected, no expression was detectable in the Δ*recA* strain when a primer set located in the deleted region was used. The apparent expression level in the wild-type strain appeared to differ between the two primer sets, but this was not statistically significant (*p* > 0.05, Students *t*-test). The observed expression level for Rv2735c was at a lower level than *recA* or *recX*. It has been observed previously that downstream genes in an operon are expressed at a lower level than promoter proximal genes, including in actinomycetes.^32–34^ The most significant finding was that expression of *recX* and Rv2735c was still detectable, albeit at a reduced level, in the Δ*recA* strain (Figure 2). This raised the possibility that this residual expression might arise from a promoter other than those already characterised upstream of *recA*.

An alternative explanation could be that the residual expression of *recX* in the Δ*recA* strain might originate from transcriptional read-through from the *recA* promoters owing to incomplete termination in the hygromycin resistance cassette. To assess this possibility, we compared the ability of RNA isolated from the Δ*recA* strain with that from the wild-type parental strain to act as template in RT-PCR reactions when one primer was located in *recA* and the other was in *recX* or Rv2735c. The precise locations of the primers within the *recA*-*recX* sequence are shown in Supplementary figure 1 along with other key features. When wild-type RNA was used, we obtained a product of the expected size for each primer pair combination used following reverse transcription, but not if the reverse transcription step was omitted (Figure 1). However, none of the primer combinations that included a primer located in *recA* yielded an RT-PCR product when RNA isolated from the Δ*recA* strain was used (Figure 1). In contrast, a product was formed when a primer in *recX* was used in combination with one in Rv2735c (Figure 1), demonstrating that the RNA isolated from the mutant strain was capable of being reverse-transcribed into cDNA that could act as a substrate for PCR. Although the binding site for one of the primers used in *recA* would have been removed in the deletion strain, for one of the other primers in *recA* the binding site was upstream of the deletion, while another would bind immediately downstream of the deletion. Therefore, the lack of RT-PCR product formation for these two primer pairs with RNA from the Δ*recA* strain argues against both transcriptional read-through from the *recA* promoters and expression originating from a promoter within the hygromycin resistance fragment.

### 3.3 Localisation of promoter activity

**Figure 3:**
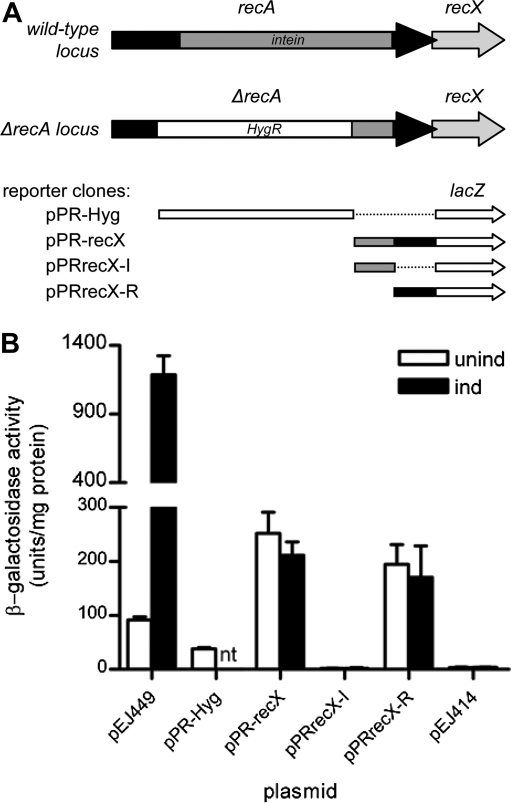
Identification of promoter activity upstream of *recX*. (A) Schematic showing the arrangement of the *recA* locus in the wild-type and in the Δ*recA* strain at the top, with the various fragments cloned in the *lacZ* reporter vector aligned with the corresponding part of the locus below. The RecA coding sequence is shown in black, the intein sequence in dark grey, and the *recX* sequence in light grey, with the hygromycin resistance fragment indicated by a white box and the *lacZ* transcriptional reporter gene by a white arrow. (B) Promoter activity conferred on a *lacZ* reporter gene by transcriptional fusion with the DNA fragments indicated in (A), or with the *recA* P1 promoter in the positive control pEJ449. pPR-Hyg contains the fragment encoding resistance to hygromycin in the the *recA* mutant, pPRrecX contains the 3′end of *recA* from the end-point of the deletion in the *recA* mutant to the beginning of the RecX coding sequence, pPRrecX-I contains the intein-encoding part of the 3′end of *recA* and pPRrecX-R contains the RecA-encoding part of the 3′end of *recA*. pEJ414 is the vector control. β-galactosidase activity was determined using untreated cultures of *M. tuberculosis* (white bars) or cultures exposed to 0.02 μg/ml mitomycin C for 24 h (black bars). Values are the means from three independent cultures, each of which was assayed in duplicate; error bars show standard deviations.

The results described above suggested that the residual expression of *recX* observed in the Δ*recA* strain most likely arose from a promoter located in the remaining part of the *recA* gene downstream of the deletion/insertion, although the possibility of a promoter in the hygromycin resistance fragment driving expression was not completely excluded. To distinguish between these possibilities, we made two transcriptional fusions to the reporter gene *lacZ*. The first, pPR-Hyg, contained the DNA fragment conferring resistance to hygromycin present in the Δ*recA* strain and the second, pPRrecX, contained the 3′-end of *recA* from the end-point of the deletion in the Δ*recA* strain to the beginning of the RecX coding sequence. Following transformation into wild-type H37Rv bacteria, we were surprised to detect promoter activity from both fragments (Figure 3), although the activity residing within the 3′-end of *recA* was substantially greater than that from the hygromycin resistance fragment.

As part of the DNA remaining at the 3′-end of *recA* in the deletion strain corresponds to the intein, we wanted to know if the promoter in this region was located in the intein-encoding DNA or in the RecA-encoding sequence. We, therefore, subdivided this region and made two further *lacZ* reporter constructs, one pPRrecX-I containing just the intein sequence and the other pPRrecX-R just the RecA sequence. Analysis of these clones in *M. tuberculosis* H37Rv clearly demonstrated that the promoter activity resided in the RecA-encoding DNA only (Figure 3).

To assess if the promoter identified at the 3′-end of *recA* was DNA damage-inducible, we compared the expression levels obtained from the relevant reporter clones following exposure to the DNA damaging agent mitomycin C (0.02 μg/ml for 24 h) with an unexposed control. The presence or absence of mitomycin C did not alter the expression levels obtained (Figure 3). In contrast, the *recA* P1 promoter contained within pEJ449 used as a positive control was clearly inducible, as previously demonstrated^22^ (Figure 3). Thus, the promoter within the 3′-end of *recA* is constitutive.

### 3.4 Identification of promoter elements

In order to confirm the presence of promoters, we sought to identify the promoter elements and then assess the effect of introducing mutations in the −10 regions. To facilitate the identification of promoter elements, we first mapped the transcript start sites by 5′ RACE.

This revealed that in the reporter clone containing the hygromycin resistance fragment the transcript start site was actually located within vector sequence between the insert and the *lacZ* gene and that the −10 region was located at the junction of the insert and vector. It appears that in constructing this clone, combining vector sequence with very limited homology to the SigA −10 consensus (normally incapable of driving expression of the reporter gene as demonstrated by assays on the empty vector in Figure 3), with insert sequence with a reasonable match to the SigA −35 consensus created a weak artificial promoter. Thus, the promoter activity apparently originating from the hygromycin resistance fragment is an artefact of cloning, and was not investigated further.

**Figure 4:**
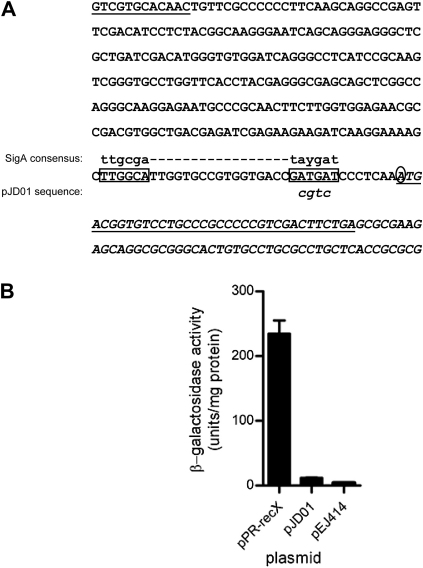
The location of the *recX* promoter at the 3′-end of *recA* and the effect of mutation of the −10 region. (A) The sequence of the 3′-region of *recA* and the 5′-region of *recX* showing the location of the *recX* promoter at the 3′-end of *recA* in relation to the intein and the two coding regions. The 3′-end of the intein is underlined with a dotted line. The *recX* sequence is in italics. The region of overlap of *recX* with *recA* is underlined, with the start codon of *recX* and the stop codon of *recA* double underlined. The −35 and −10 promoter elements are boxed and the transcription start site for this promoter is circled. The SigA consensus sequence is shown in lower case above the promoter elements. The bases replacing the native −10 motif in the mutated reporter clone pJD01 are indicated in italic lower case below the −10 motif of the wild-type sequence. (B) Effect of introducing the sequence change shown in (A) on *recX* promoter activity, compared with the vector pEJ414. β-galactosidase activity was determined from cultures of *M. tuberculosis* grown to OD_600_ of 0.6; values are the means from three independent cultures, each of which was assayed in duplicate and the error bars show standard deviations.

The transcript start site for *recX* was mapped to the A corresponding to the first base of the ATG initiation codon. Upstream of this, motifs (TTGGCA-N_17_-GATGAT) resembling both the −35 and −10 consensus sequences for SigA (TTGACW-N_17_-TATAMT) were identifiable at the appropriate locations (Figure 4). To confirm that the promoter had been correctly identified, we introduced a mutation in the −10 motif, changing the central ATGA to CGTC in pJD01. When assayed in *M. tuberculosis* H37Rv the corresponding promoter activity was reduced to levels comparable with the vector control (Figure 4). Thus, the presence of an active promoter within the coding sequence of *recA* was verified.

### 3.5 The *recX* promoter is not conserved in all mycobacteria

**Figure 5:**
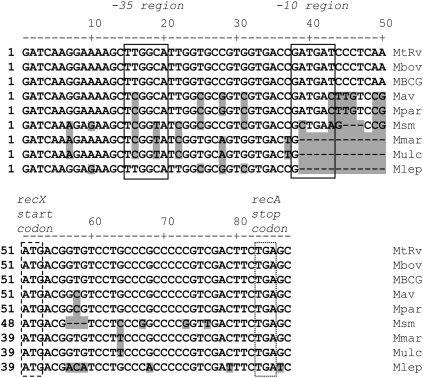
Extent of conservation of the *recX* promoter amongst mycobacterial species. Alignment of the 3′-end of *recA* and the 5′-end of *recX* from *M. tuberculosis* H37Rv (MtRv), *M*. *bovis* AF2122/97 (Mbov), *M. bovis* BCG str. Pasteur 1173P2 (MBCG), *M. avium* 104 (Mav), *M. avium* subsp. *paratuberculosis* K-10 (Mpar), *M. smegmatis* mc^2^155 (Msm), *M. marinum* ATCC BAA-535 (Mmar), *M. ulcerans* Agy99 (Mulc), and *M. leprae* TN (Mlep). The locations of the −10 and −35 motifs are boxed with solid lines and labeled above the sequences, the RecX start codon (position 51–53 in MtRv) is boxed with a dashed line and labeled, and the RecA stop codon is boxed with a dotted line and labeled. Bases that differ from the *M. tuberculosis* sequence are shaded. The −10 motif is absent in *M. marinum*, *M. ulcerans* and *M. leprae*, and has a potentially inactivating base change in *M. smegmatis*.

In order to gain an indication as to how widespread the *recX* promoter internal to *recA* is, we aligned the sequences of the 3′-end of *recA* and the 5′ end of *recX* from a variety of mycobacterial species (Figure 5). Not surprisingly, the sequence was completely conserved in *Mycobacterium bovis* and *M. bovis* BCG. Highly conserved motifs were present in *M. avium* and *M. avium* subsp. *paratuberculosis*, differing from the *M. tuberculosis* sequence by only a single base in each of the −10 and −35 motifs. In contrast, the −10 region was completely absent in *Mycobacterium marinum*, *M. ulcerans* and *M. leprae*. In *M. smegmatis* although the corresponding motifs were recognisable, one of the base changes in the −10 region (A to C at −12) would be likely to inactivate the promoter.

To test if this is the case, we cloned the corresponding region from *M. smegmatis* into the *lacZ* transcriptional reporter vector, creating pJD05. When assayed in *M. smegmatis*, the expression driven by this sequence (3.0 ± 0.4 units/mg protein) did not differ significantly (*p* > 0.05, Students *t*-test) from the background level seen with the vector control (2.2 ± 0.4 units/mg protein). Thus, *recX* does not have an additional promoter other than those upstream of *recA* in *M. smegmatis*.

### 3.6 What is the significance of the *recX* promoter in *M. tuberculosis*?

**Figure 6:**
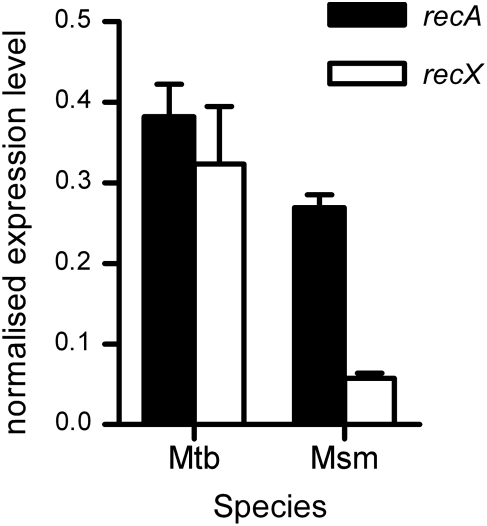
Comparison of the expression level of *recX* relative to *recA* in *M. tuberculosis* and *M. smegmatis*. qRT-PCR was performed as described in the Methods using RNA isolated from wild-type *M. tuberculosis* (Mtb) or *M. smegmatis* (Msm). Expression levels of *recA* (black bars), using primer set RecA_up for M. tuberculosis, and *recX* (white bars) were normalised to that of the housekeeping gene *sigA* for each species. Values are the means from three independent cultures, each of which was assayed in triplicate; error bars show standard deviations.

The presence of an additional promoter for *recX* in *M. tuberculosis* compared with *M. smegmatis* would be expected to result in a higher *recX* expression level under non-DNA damaging conditions. To assess if this is the case we compared the expression of *recX* with that of *recA* in wild-type H37Rv *M. tuberculosis* and *M. smegmatis* by qRT-PCR, normalising to *sigA*. In *M. tuberculosis*, there was no significant difference (*p* > 0.05, Students *t*-test) in the expression levels of the two genes (Figure 6). In contrast, in *M. smegmatis recX* was expressed at a significantly lower level (*p* < 0.01, Students *t*-test) than *recA* (Figure 6), although the level of *recA* was similar in the two species. Thus, the ratio of *recX* to *recA* in *M. smegmatis* was a quarter of the corresponding value for *M. tuberculosis* (0.21 compared with 0.84).

### 3.7 How common are promoters internal to genes in *M. tuberculosis*?

We wondered if promoters were also present within the 3′-ends of other *M. tuberculosis* coding sequences. To gain some insight into this question, we took a bioinformatic approach. First we searched the *M. tuberculosis* genome sequence for sequences matching the SigA consensus that were located no more than 200 bp upstream of genes, permitting a maximum of 3 mismatches in total, of which no more than 2 could be in a single motif (either the −10 or −35 motif). We identified 231 regions matching these criteria. We then looked to see how many of these were located upstream of the stop codon of the preceding gene. We found that 84 were within 200 bp (Supplementary table 1), and 44 were within 100 bp, of such a stop codon, representing 36% and 20% of predicted promoters respectively. For comparison, we performed the equivalent searches on the *E. coli* genome but using the σ^70^ consensus sequence. This revealed the presence of 457 predicted promoters in the 200 bp regions upstream of genes. However, only 49 of these were within 200 bp of the stop codon of the preceding gene (Supplementary table 2), and only 22 within 100 bp, representing 11% and 5% of the predicted promoters respectively. Thus, although almost twice as many promoters were predicted within 200 bp upstream of genes in *E. coli* than in *M. tuberculosis*, a much smaller proportion of these were located upstream of the stop codon of the preceding gene, despite the gene density in the two organisms being very similar.^18,35^ This suggests that promoters internal to coding regions may be more common in *M. tuberculosis* than in some other bacteria.

## 4 Discussion

In this study we have demonstrated that *M. tuberculosis recX* is expressed from a constitutive promoter located within the coding sequence of *recA*, the gene immediately upstream of *recX*, in addition to being co-transcribed with *recA* from its two DNA-damage inducible promoters. The occurrence of promoters internal to coding sequences responsible for transcription of downstream genes in bacteria has only recently been recognised.^36^ Our bioinformatic analysis suggests that promoters so located may be more common in *M. tuberculosis* than in some other bacteria. The development of genomic RNA sequencing approaches is now facilitating the identification of such promoters, such that over 400 internal transcription start sites have been identified in the pathogen *Helicobacter pylori*.^37^ It will be interesting to see what this approach reveals in the case of *M. tuberculosis*.

Transcription of *recX* from the promoter internal to *recA* initiated at the A of the *recX* ATG codon. Although relatively uncommon, a few examples of such leaderless mRNAs have been described previously from *M. tuberculosis*: *purC*,^38^ *oxyR*,^39^ *eis*^40^ and *lexA*.^41,42^ Leaderless mRNAs interact preferentially with 70S ribosomes in bacteria^43^ and similar findings in eukaryotes have led to the suggestion that this type of mRNA might be evolutionary ancient.^44^ Cross-linking studies have revealed that leaderless mRNAs are recognised by the ribosome initially via the AUG codon; indeed, the addition of an AUG triplet to a random RNA sequence was found to confer ribosome binding.^45^ The presence of initiator tRNA conferred greater stability on the interaction, as revealed by gel shift and toeprinting assays.^45^ Translation of leaderless mRNAs has also been shown to be preferentially stimulated by initiation factor 2 (IF2),^46^ and the ratio of IF2 to IF3 can modify the efficiency of translation initiation on such RNAs,^47^ potentially providing a means of modulating translation of leaderless mRNAs in response to conditions causing a variation in the availability of these components of the translational machinery.

The expression of the reporter gene *lacZ* driven by the *recX* promoter (ca. 200 units) was at a comparable level to that conferred by either of the *recA* promoters under non-DNA damaging conditions (P1 ca. 100 units and P2 ca. 230 units^48^) when present on fragments of similar size. Thus, it can be expected to make a significant contribution to the expression of *recX* in the absence of DNA damage. Intriguingly, this *recX* promoter is missing in some other mycobacterial species (*M. marinum*, *M. ulcerans* and *M. leprae*) and is not active in *M. smegmatis* owing to base changes within the promoter elements. At present it is not clear whether *M. avium* and *M. avium* subsp. *paratuberculosis* possess an active promoter in this region. As would be expected from the observations described above, the expression of *recX* relative to that of *recA* was lower in *M. smegmatis* than in *M. tuberculosis*.

RecX protein interacts with RecA to modulate its activities.^9–11^ Thus, the elevated ratio of RecX to RecA in *M. tuberculosis* compared with *M. smegmatis* may influence RecA function. RecX inhibits the ATPase and strand-exchange activities of RecA *in vitro*, and so may affect DNA repair processes in the cell. Indeed, the lack of *recX* has been reported to have a small effect on survival following UV irradiation in *E. coli*.^9^ RecX also inhibits the ability of RecA to stimulate cleavage of the repressor protein LexA *in vitro*^11^ and, when overexpressed, in *E. coli* cells.^9^ This latter property results in a reduced rate of induction of LexA-regulated genes in response to DNA damage under the tested conditions. Thus, it is possible that higher basal expression of RecX relative to RecA in *M. tuberculosis* compared with *M. smegmatis* may contribute to the previously observed slower kinetics of induction of the SOS response in *M. tuberculosis*.^23^ However, it should be remembered that once induction has occurred, the expression of *recX* will be dominated by the elevated activity of the two *recA* promoters which are both induced by DNA damage.^22,48^ To definitively assess the biological significance of the *recX* promoter located within the *recA* coding sequence it would be necessary to introduce point mutations that inactivate this promoter and compare the resulting phenotype with a strain having the wild-type sequence. However, this is problematical as the point mutation most likely to inactivate the promoter (A to C at −12) would also result in an amino acid change in RecA (aspartic acid to alanine in the above example) that could affect its activity. The only silent point mutation in terms of coding sequence (T to C at −11) seems less likely to inactivate the promoter, although this could be tested by introducing the base change into a transcriptional fusion plasmid such as pPRrecX.

## Funding:

This work was funded by the UK Medical Research Council (programme number U1175 32056).

## Competing interests

None declared.

## Ethical approval

Not required.

## Figures and Tables

**Table 1:** Plasmids used in this study.

Plasmid	Construction/relevant characteristics
pCR4-Blunt-TOPO	TOPO cloning vector (Invitrogen)
pEJ414	*lacZ* transcriptional reporter plasmid with mycobacteriophage L5 attachment site and integrase genes. Kan^R^^23^
pEJ449	*recA* P1 promoter in pEJ414^22^
pPR-Hyg	1780 bp fragment encompassing HygR cassette in Δ*recA* strain in pEJ414 (PCR fragment from Hyg_PrF and Hyg_PrR)
pPRrecX	580 bp fragment 3′-end *M. tuberculosis recA* coding sequence upstream of *recX* in pEJ414 (PCR fragment from recA_F2 and recX_PrR)
pPRrecX-I	314 bp fragment equivalent of first 314 bp of cloned fragment in pPR recX in pEJ414 (PCR fragment from recA_F2 and Intein_R)
pPRrecX-R	266 bp fragment equivalent of last 265 bp of cloned fragment in pPR recX in pEJ414 (PCR fragment from recX_Pr2F and recX_PrR)
pJD01	ATGA to CGTC mutation in −10 region of pPRrecX (site-directed mutagenesis using X2SDMF and X2SDMR)
pJD05	285 bp fragment 3′-end *M. smegmatis recA* coding sequence upstream of *recX* in pEJ414 (PCR fragment from Msm_recX_PrF and Msm_recX_PrR)

**Table 2:** Primers used in this study.

Name	Sequence (5′→3′)	Position relative to translation start site of gene indicated.
Plasmid construction^a^		
recA_F2	*CTGG**TCTA*GAGATGACCGATGCCGTGCTGAATTATC	−579 to −553 *recX*
recX_PrR	*GACGCGGCCGC*TTTGAGGGATCATCGGTCA	−18 to +1 *recX*
Intein_R	*GGGAAGCTT*GTTGTGCACGACAACCCCTTCGG	−288 to −266 *recX*
recX_Pr2F	*GTGTCTAGA*TGTTCGCCCCCCTTCAAGCAGG	−265 to −244 *recX*
Hyg_PrF	*CACTCTAGA*CGATCTGAGCTTGCATGCCTGC	n/a
Hyg_PrR	*GCAAAGCTT*GGTCATCTCGATCTGGCTCG	n/a
Msm_recX_PrF	*GGTCTAGA*CGCCGTTCAAGCAG	−255 to −242 *recX*
Msm_recX_PrR	*CGAAGCTT*CAGAAGTCAACCGG	+18 to +31 *recX*

RecA operon co-transcription∗		
recX_F1	GCACCCGCGCCGAGTTAGC	+89 to +107 *recX*
recA_F1	TCGCGGATGCCCTGGATGACAAAT	−426 to −403 *recX*
recA_F2	*CTGGTCTA*GAGATGACCGATGCCGTGCTGAATTATC	−579 to −553 *recX*
Rv2735c_R1	AGTTCCGCGTTCGTGCCCTTCA	+506 to +527 *Rv2735c*
recA_F3	*AAGTCTAGA*TGCCTCGCAGAGGGCACTCGG	+754 to +774 *recA*
recA_F4	GCTCAGGCCGCCGGTGGTGTTG	+250 to +271 *recA*
RecX_R1	CGTTCGCCCGCCTGGACTGA	+216 to +235 *recX*

5′ RACE		
GSP_recX	GCTCGGCGGCCAGCTCGGCGATAAC	+481 to +505 *recX*
GSP_recX_Nest	GCTTTTCCGCCCGCCCCCGTTCG	+333 to +355 *recX*
GSP_Hyg	TGTGCGGCGAGTTGCGTGA	n/a
GSP_Hyg_Nest	GTCGTCGTCCCCTCAAACTGG	n/a

qRT-PCR		
SigA_F	TCGGTTCGCGCCTACCT	+679 to +695 *sigA*
SigA_R	TGGCTAGCTCGACCTCTTCCT	+731 to +751 *sigA*
recA_up_F	ATCGAGAAGAGTTACGGCAAAGG	+52 to +74 *recA*
recA_up_R	GCCCAGGGCCACGTCTA	+143 to +159 *recA*
RecA_del_F	ACCGGCGCGCTGAATA	+538 to +553 *recA*
RecA_del_R	CGCGGAGCTGGTTGATG	+576 to +592 *recA*
RecX_F	GCACCCGCGCCGAGTTAG	+89 to +106 *recX*
RecX_R	GGCCAGCCGATCCAATACCC	+152 to +171 *recX*
2735c_F	TAACCCGCTTGCCTCTGAA	+228 to +246 *Rv2735c*
2735c_R	ACCTACCGTCACCGGGAAAG	+270 to +289 *Rv2735c*
Msm_sigA_F	CCCACCGGGAATTCGTAAG	−34 to −16 *sigA*
Msm_sigA_R	TTGCCGGGCTTGCCTT	+13 to +28 *sigA*
Msm_recA_F	TGACCGGCGCGTTGA	+539 to +553 *recA*
Msm_recA_R	CGGAGCTGGTTGATGAAGATC	+573 to +593 *recA*
Msm_recX_F	TGCGCCGCGAACGT	+412 to +425 *recX*
Msm_recX_R	CCTGCGGGTGACCTTGAC	+450 to +467 *recX*

Site-directed mutagenesis†		
X2SDM_F	CATTGGTGCCGTGGTGACCGcgtcTCCCTCAAGCGGCCGCCACG	n/a
X2SDM_R	CGTGGCGGCCGCTTGAGGGAgacgCGGTCACCACGGCACCAATG	n/a

∗ Underlined bases indicate restriction sites added to primers to facilitate cloning of PCR products and bases in italics indicate bases that are not homologous to the native sequence.

† Bases in lower case indicate bases different from the wild-type sequence.
