# Supplementary material for: Enhanced expression of recX in Mycobacterium tuberculosis owing to a promoter internal to recA
Source: Tuberculosis (Edinb). 2011 Mar;91(2):127–35. doi: 10.1016/j.tube.2010.11.002 (PMC3062782; doi:10.1016/j.tube.2010.11.002)
Supplement: Supplementary file 1 [file mmc1.doc]

Legend to Supplementary Figure

The sequence of *recA* and *recX* showing the location of the promoter at the 3’-end of *recA* in relation to the deleted region and the intein. The sequence missing in the deletion mutant is shown in lower case, the intein sequence is underlined, and the *recX* sequence is in italics with the region of overlap with *recA* indicated by the wavy underlining. The -35 and -10 promoter elements identified in this study are boxed and the transcription start site for this promoter is circled. The annealing sites for the forward primers used for RT-PCR are shown in red or orange, and that for the reverse primer in *recX* is in blue, with the primer names shown above the highlighted sequences. Similarly, the annealing sites for the primers used to generate the PCR products cloned as transcriptional fusions are shown in orange or green for the forward primers and in purple for the reverse primers, with the primer names shown above the highlighted sequences. The location of the reverse primer in Rv2735c used for RT-PCR is not shown as the sequence of Rv2735c is not included. Some primers included additional bases containing restriction sites to facilitate cloning that are not homologous to the native sequence.
