# Supplementary material for: Enhanced expression of recX in Mycobacterium tuberculosis owing to a promoter internal to recA
Source: Tuberculosis (Edinb). 2011 Mar;91(2):127–35. doi: 10.1016/j.tube.2010.11.002 (PMC3062782; doi:10.1016/j.tube.2010.11.002)
Supplement: Supplementary file 2 [file mmc2.ppt]

## Slide 1
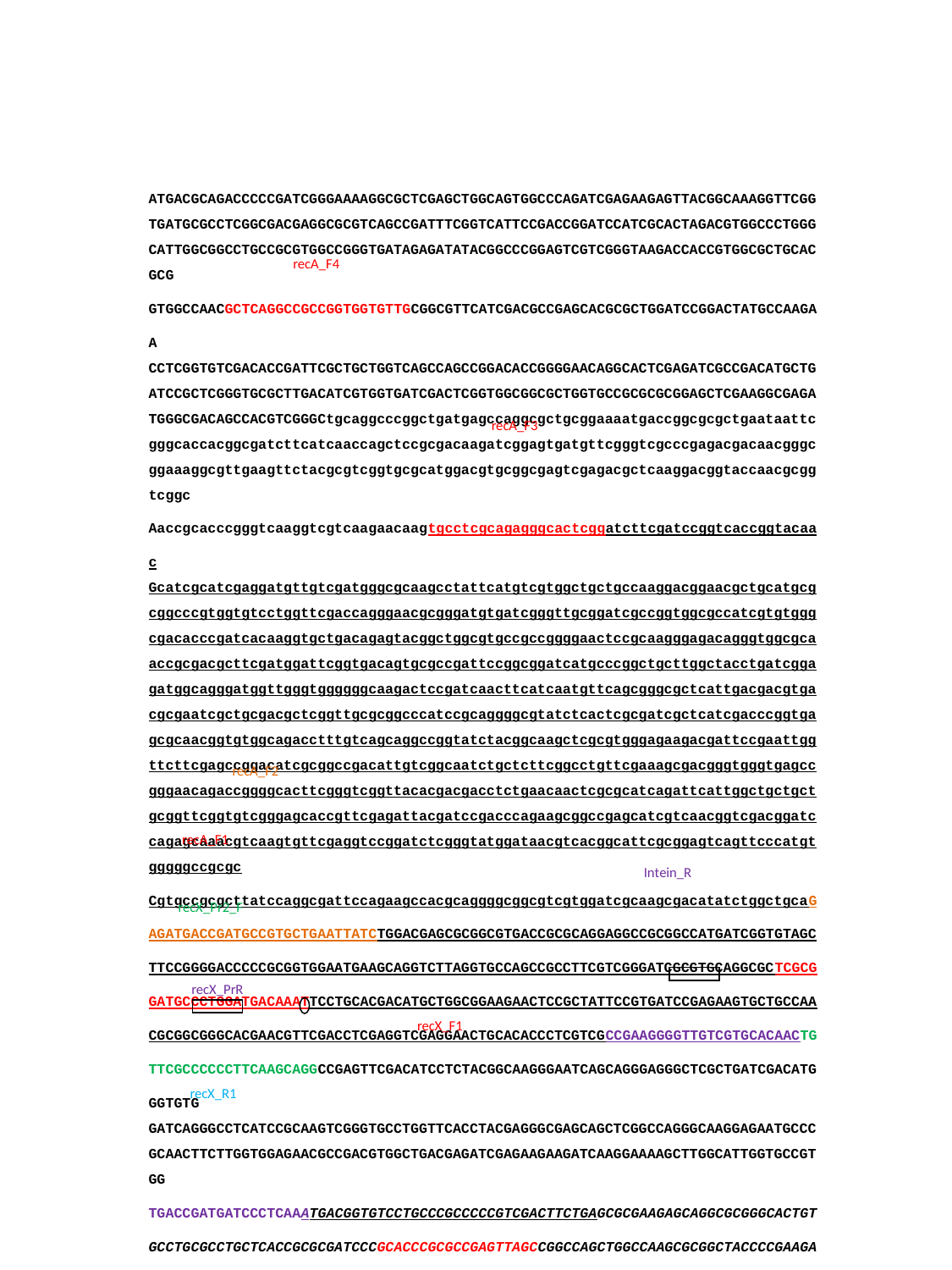

ATGACGCAGACCCCCGATCGGGAAAAGGCGCTCGAGCTGGCAGTGGCCCAGATCGAGAAGAGTTACGGCAAAGGTTCGGTGATGCGCCTCGGCGACGAGGCGCGTCAGCCGATTTCGGTCATTCCGACCGGATCCATCGCACTAGACGTGGCCCTGGGCATTGGCGGCCTGCCGCGTGGCCGGGTGATAGAGATATACGGCCCGGAGTCGTCGGGTAAGACCACCGTGGCGCTGCACGCG
GTGGCCAACGCTCAGGCCGCCGGTGGTGTTGCGGCGTTCATCGACGCCGAGCACGCGCTGGATCCGGACTATGCCAAGAA
CCTCGGTGTCGACACCGATTCGCTGCTGGTCAGCCAGCCGGACACCGGGGAACAGGCACTCGAGATCGCCGACATGCTGATCCGCTCGGGTGCGCTTGACATCGTGGTGATCGACTCGGTGGCGGCGCTGGTGCCGCGCGCGGAGCTCGAAGGCGAGATGGGCGACAGCCACGTCGGGCtgcaggcccggctgatgagccaggcgctgcggaaaatgaccggcgcgctgaataattcgggcaccacggcgatcttcatcaaccagctccgcgacaagatcggagtgatgttcgggtcgcccgagacgacaacgggcggaaaggcgttgaagttctacgcgtcggtgcgcatggacgtgcggcgagtcgagacgctcaaggacggtaccaacgcggtcggc
Aaccgcacccgggtcaaggtcgtcaagaacaagtgcctcgcagagggcactcggatcttcgatccggtcaccggtacaac
Gcatcgcatcgaggatgttgtcgatgggcgcaagcctattcatgtcgtggctgctgccaaggacggaacgctgcatgcgcggcccgtggtgtcctggttcgaccagggaacgcgggatgtgatcgggttgcggatcgccggtggcgccatcgtgtgggcgacacccgatcacaaggtgctgacagagtacggctggcgtgccgccggggaactccgcaagggagacagggtggcgcaaccgcgacgcttcgatggattcggtgacagtgcgccgattccggcggatcatgcccggctgcttggctacctgatcggagatggcagggatggttgggtggggggcaagactccgatcaacttcatcaatgttcagcgggcgctcattgacgacgtgacgcgaatcgctgcgacgctcggttgcgcggcccatccgcaggggcgtatctcactcgcgatcgctcatcgacccggtgagcgcaacggtgtggcagacctttgtcagcaggccggtatctacggcaagctcgcgtgggagaagacgattccgaattggttcttcgagccggacatcgcggccgacattgtcggcaatctgctcttcggcctgttcgaaagcgacgggtgggtgagccgggaacagaccggggcacttcgggtcggttacacgacgacctctgaacaactcgcgcatcagattcattggctgctgctgcggttcggtgtcgggagcaccgttcgagattacgatccgacccagaagcggccgagcatcgtcaacggtcgacggatccagagcaaacgtcaagtgttcgaggtccggatctcgggtatggataacgtcacggcattcgcggagtcagttcccatgtgggggccgcgc
CgtgccgcgcttatccaggcgattccagaagccacgcaggggcggcgtcgtggatcgcaagcgacatatctggctgcaGAGATGACCGATGCCGTGCTGAATTATCTGGACGAGCGCGGCGTGACCGCGCAGGAGGCCGCGGCCATGATCGGTGTAGCTTCCGGGGACCCCCGCGGTGGAATGAAGCAGGTCTTAGGTGCCAGCCGCCTTCGTCGGGATCGCGTGCAGGCGCTCGCGGATGCCCTGGATGACAAATTCCTGCACGACATGCTGGCGGAAGAACTCCGCTATTCCGTGATCCGAGAAGTGCTGCCAACGCGGCGGGCACGAACGTTCGACCTCGAGGTCGAGGAACTGCACACCCTCGTCGCCGAAGGGGTTGTCGTGCACAACTGTTCGCCCCCCTTCAAGCAGGCCGAGTTCGACATCCTCTACGGCAAGGGAATCAGCAGGGAGGGCTCGCTGATCGACATGGGTGTG
GATCAGGGCCTCATCCGCAAGTCGGGTGCCTGGTTCACCTACGAGGGCGAGCAGCTCGGCCAGGGCAAGGAGAATGCCCGCAACTTCTTGGTGGAGAACGCCGACGTGGCTGACGAGATCGAGAAGAAGATCAAGGAAAAGCTTGGCATTGGTGCCGTGG
TGACCGATGATCCCTCAAATGACGGTGTCCTGCCCGCCCCCGTCGACTTCTGAGCGCGAAGAGCAGGCGCGGGCACTGTGCCTGCGCCTGCTCACCGCGCGATCCCGCACCCGCGCCGAGTTAGCCGGCCAGCTGGCCAAGCGCGGCTACCCCGAAGACATCGGCAACCGGGTATTGGATCGGCTGGCCGCCGTTGGCCTGGTGGATGACACCGACTTCGCCGAACAATGGGTTCAGTCCAGGCGGGCGAACGCAGCAAAGAGCAAGCGCGCGTTGGCTGCCGAGCTGCACGCCAAGGGCGTCGACGACGACGTGATCAC
CACGGTGCTCGGGGGCATCGACGCCGGTGCCGAACGGGGGCGGGCGGAAAAGCTGGTACGGGCCAGGCTGCGGCGGGAGGTGCTGATCGACGACGGCACCGACGAAGCGCGGGTGAGCCGCAGGCTGGTGGCGATGTTGGCGCGCCGTGGGTACGGCCAGACCTTGGCGTGCGAGGTGGTTATCGCCGAGCTGGCCGCCGAGCGGGAGCGCCGACGCGTCTAA
recA_F4
recA_F3
recA_F2
recA_F1
Intein_R
recX_Pr2_F
recX_PrR
recX_F1
recX_R1
